# Associations of PM_2.5_ Constituents and Sources with Hospital Admissions: Analysis of Four Counties in Connecticut and Massachusetts (USA) for Persons ≥ 65 Years of Age

**DOI:** 10.1289/ehp.1306656

**Published:** 2013-11-08

**Authors:** Michelle L. Bell, Keita Ebisu, Brian P. Leaderer, Janneane F. Gent, Hyung Joo Lee, Petros Koutrakis, Yun Wang, Francesca Dominici, Roger D. Peng

**Affiliations:** 1 School of Forestry and Environmental Studies, Yale University, New Haven, Connecticut, USA; 2 Yale Center for Perinatal, Pediatric and Environmental Epidemiology, School of Public Health, Yale University, New Haven, Connecticut, USA; 3 Department of Environmental Health, and; 4 Department of Biostatistics, School of Public Health, Harvard University, Boston, Massachusetts, USA; 5 Department of Biostatistics, Johns Hopkins Bloomberg School of Public Health, Baltimore, Maryland, USA

## Abstract

Background: Epidemiological studies have demonstrated associations between short-term exposure to PM_2.5_ and hospital admissions. The chemical composition of particles varies across locations and time periods. Identifying the most harmful constituents and sources is an important health and regulatory concern.

Objectives: We examined pollutant sources for associations with risk of hospital admissions for cardiovascular and respiratory causes.

Methods: We obtained PM_2.5_ filter samples for four counties in Connecticut and Massachusetts and analyzed them for PM_2.5_ elements. Source apportionment was used to estimate daily PM_2.5_ contributions from sources (traffic, road dust, oil combustion, and sea salt as well as a regional source representing coal combustion and other sources). Associations between daily PM_2.5_ constituents and sources and risk of cardiovascular and respiratory hospitalizations for the Medicare population (> 333,000 persons ≥ 65 years of age) were estimated with time-series analyses (August 2000–February 2004).

Results: PM_2.5_ total mass and PM_2.5_ road dust contribution were associated with cardiovascular hospitalizations, as were the PM_2.5_ constituents calcium, black carbon, vanadium, and zinc. For respiratory hospitalizations, associations were observed with PM_2.5_ road dust, and sea salt as well as aluminum, calcium, chlorine, black carbon, nickel, silicon, titanium, and vanadium. Effect estimates were generally robust to adjustment by co-pollutants of other constituents. An interquartile range increase in same-day PM_2.5_ road dust (1.71 μg/m^3^) was associated with a 2.11% (95% CI: 1.09, 3.15%) and 3.47% (95% CI: 2.03, 4.94%) increase in cardiovascular and respiratory admissions, respectively.

Conclusions: Our results suggest some particle sources and constituents are more harmful than others and that in this Connecticut/Massachusetts region the most harmful particles include black carbon, calcium, and road dust PM_2.5_.

Citation: Bell ML, Ebisu K, Leaderer BP, Gent JF, Lee HJ, Koutrakis P, Wang Y, Dominici F, Peng RD. 2014. Associations of PM_2.5_ constituents and sources with hospital admissions: analysis of four counties in Connecticut and Massachusetts (USA) for persons ≥ 65 years of age. Environ Health Perspect 122:138–144; http://dx.doi.org/10.1289/ehp.1306656

## Introduction

Associations between airborne particulate matter (PM) and health are well established (Pope and Dockery 2006), including evidence of higher risk associated with smaller particles with an aerodynamic diameter of ≤ 2.5 μm (PM_2.5_). Several countries regulate PM_2.5_ (e.g., the United States, the United Kingdom, Taiwan), and the World Health Organization (WHO) has established health-based guidelines. Increasing scientific evidence suggests that particles differ in toxicity. This hypothesis is consistent with known heterogeneity in particles’ chemical composition (Bell et al. 2007). For example, sulfate constitutes a higher fraction of PM_2.5_ in the eastern United States than in the western United States. Composition of PM_2.5_ in Seoul, Korea, is more similar to PM_2.5_ in the western United States than PM_2.5_ in the eastern United States (Son et al. 2012). Variations in composition may affect health risks and explain why effect estimates for PM_2.5_, measured by total mass, differ by location.

The Health Effects Institute (HEI), a National Academies of Sciences committee, and the WHO identified the study of health effects of the particle mixture as a critical research need (HEI 2002; National Research Council 2004; WHO 2007). Evidence on which particles are most harmful would inform effective policies by allowing stricter control of the most harmful agents and could aid understanding of biological pathways, which may differ by constituents or health outcomes. Multiple biologically plausible mechanisms have been demonstrated or hypothesized [e.g., systematic inflammation, vascular function (Brook et al. 2010)] although physiological responses to different PM_2.5_ constituents and sources are not fully understood.

Many epidemiological studies use existing ambient monitoring data from regulatory agencies to estimate air pollution exposure. This approach is cost effective and can cover large populations and time periods. Limited availability of PM_2.5_ constituent data, compared with data for total PM_2.5_, limits research on particulate composition and health. National U.S. monitoring networks for PM_2.5_ constituents began operation in 1999, with many monitors beginning in 2000. The U.S. Environmental Protection Agency (EPA) has monitored PM_2.5_ since 1997, with many monitors starting in 1999. The PM_2.5_ monitoring network is more extensive, with 1,387 active monitors in the continental United States, whereas the PM_2.5_ Chemical Speciation Network has 192 monitors (U.S. EPA 2012). Additional monitors with chemical speciation are available for rural sites through the Interagency Monitoring of Protected Visual Environments (IMPROVE) network (U.S. EPA 2013). Although data from the U.S. EPA’s constituent network are useful, data are unavailable for all time periods and locations of interest.

Several methods have been introduced to estimate pollution for times and locations without monitors, such as regional air quality modeling; however, methods to estimate complex PM_2.5_ chemical composition remain limited. Understanding the health impacts is hindered by the lack of daily measurements of constituents in national monitoring networks. To date, we are aware of only one study that has applied source apportionment methods to examine associations between PM_2.5_ sources and hospitalizations (Lall et al. 2011). In the present study, we applied an alternative approach, compared with approaches used in previous studies, to obtain additional PM_2.5_ constituent measurements. We then used these data to estimate the exposure from PM_2.5_ sources and their associated risk estimates, which are particularly relevant for policy makers because PM_2.5_ is currently regulated only on the basis of mass concentration, without regard to composition.

We used data from X-ray fluorescence elemental analysis of PM_2.5_ filters collected at five U.S. EPA monitoring sites in three counties in Connecticut and one in Massachusetts. We thus generated a new data set of PM_2.5_ chemical constituents by analyzing PM_2.5_ total mass filters for elemental composition. This new data set had almost 10 times more data (days of observation) than the U.S. EPA’s Chemical Speciation Network for the four counties. Constituent data were used in source apportionment analysis to identify particle sources. We then estimated the relative risks of cardiovascular and respiratory hospitalizations associated with short-term exposure to PM_2.5_ constituents and sources.

## Methods

*Exposure for PM_2.5_, constituents, and sources*. To estimate exposures we *a*) obtained filters used by regulatory agencies to measure PM_2.5_ total mass, *b*) analyzed those filters for PM_2.5_ elements, and *c*) used these data as inputs to source apportionment analysis. This approach generated estimates of PM_2.5_ mass, constituents, and sources for each location, for a given 24-hr day.

We acquired PM_2.5_ Teflon filter samples from the Connecticut and Massachusetts Departments of Environmental Protection for August 2000–February 2004. We considered five primary monitoring locations in four counties (see Supplemental Material, Figure S1): New Haven (in New Haven County, CT), Hartford (in Hartford County, CT), Bridgeport and Danbury (in Fairfield County, CT), and Springfield (in Hampden County, MA). Sampling occurred daily, with some missing periods, for Hartford, New Haven, and Springfield, and every third day for Bridgeport and Danbury. Because the sample days for Bridgeport and Danbury were unbiased, measurements of every third day were assumed to have no effect on central risk estimates, although it reduces sample size. Days with missing data were omitted from analysis.

The daily (midnight to midnight) PM_2.5_ filter samples were analyzed for levels of PM_2.5_ elements, using optical reflectance for black carbon (BC) (Cyrys et al. 2003; Gent et al. 2009) and X-ray fluorescence for several elements (Watson et al. 1999). Optical reflectance was performed at Harvard University and X-ray fluorescence at the Desert Research Institute in Reno, Nevada. These PM_2.5_ and constituent data were used in earlier research for other health outcomes, and more information is provided elsewhere (Bell et al. 2010; Gent et al. 2009; Lee et al. 2011).

Elemental analysis of PM_2.5_ filters produced a more extensive data set than would be available using the U.S. EPA’s constituent data. For example, the U.S. EPA’s Air Explorer (U.S. EPA 2011) PM_2.5_ constituent data from this study area and time period included data from three monitors: one each in Fairfield, New Haven, and Hampden Counties, with measurements beginning April 2002, June 2003, and December 2000, respectively. No U.S. EPA monitors assessed constituents in Hampden County. PM_2.5_ constituent data generated from PM_2.5_ filters had 9.9 times more data than the U.S. EPA’s constituent monitoring network considering all four counties, and 6.4 times more data considering the three counties with measurements in both data sets. However, the U.S. EPA’s network provides information on some constituents (e.g., nitrate, ammonium) that were unavailable for the present study.

Daily contributions of PM_2.5_ sources were estimated for each monitoring location using positive matrix factorization (PMF) (Bell et al. 2010; Norris et al. 2008; Paatero and Tapper 1994). This method identifies major PM_2.5_ sources and quantifies their daily contribution to PM_2.5_ mass and constituents. The approach estimates daily PM_2.5_ levels from each source for each site. PMF identified five sources: motor vehicles, road dust/crustal materials, oil combustion, sea salt, and regional sources related to emissions from power plants and other urban areas. We also applied PMF results in previous work, which provides more details on our methods (Bell et al. 2010).

For each county, we estimated daily levels of PM_2.5_ sources, BC, and selected constituents. We choose to analyze constituents that had been identified as potentially harmful in previous epidemiological studies (Dominici et al. 2007; Franklin et al. 2008; Lippmann et al. 2006; Ostro et al. 2007, 2008): aluminum (Al), BC, bromine (Br), calcium (Ca), chlorine (Cl), nickel (Ni), potassium (K), sulfur (S), silicon (Si), titanium (Ti), vanadium (V), and zinc (Zn). These elements were among those used in PMF analysis.

For Fairfield County, we estimated exposures using population-weighted averaging of values for the two monitoring locations in that county (Bridgeport and Danbury). Each of 209 census tracts in Fairfield County was assigned the exposure of the nearest monitor, and those exposures were averaged, weighted by each tract’s 2000 U.S. Census population. Seventy-four percent of the county’s population resided closest to the Bridgeport monitor. For other counties, we used values from the single monitor within the county. PM_2.5_ filter samples were not collected daily, so not all days had source–exposure estimates for all monitoring sites.

*Weather data*. Hourly ambient and dew point temperature data for each county were obtained from the National Atmospheric and Oceanic Administration’s (NOAA) National Climatic Data Center. These values were converted to daily levels (midnight to midnight). Daily weather values have been used extensively in previous relevant research (Samet et al. 2000a, 2000b). For each county, weather variables were estimated using data from a monitor or monitors in each county or a nearby county. For counties with multiple monitors, values from those monitors were averaged to generate county-level averages.

*Health data*. We used the Medicare beneficiary denominator file from the Centers for Medicare and Medicaid Services (CMS) to identify the at-risk population of Medicare beneficiaries ≥ 65 years of age who resided in the four counties and were enrolled in the Medicare fee-for-service plan during August 2000–February 2004. We calculated the monthly number of beneficiaries in each county to account for new enrollment and disenrollment, and extended monthly data to daily data by accounting for deaths, hospital admissions, and discharges occurring 1 day prior to an index date. We linked this time-series data with CMS Medicare inpatient claims data to identify patients discharged from acute-care hospitals. We included only emergency hospitalizations and used date of admission to calculate daily numbers of admissions. Cause of admission was determined by principal discharge diagnosis code according to *International Classification of Diseases, Ninth Revision, Clinical Modification* (ICD-9-CM; National Center for Health Statistics 2006). Analysis was conducted separately for respiratory disease (chronic obstructive pulmonary disease [ICD-9-CM codes 490–492] and respiratory tract infection [codes 464–466, 480–487]) and cardiovascular disease (heart failure [code 428], heart rhythm disturbances [codes 426–427], cerebrovascular events [codes 430–438], ischemic heart disease [codes 410–414, 429], and peripheral vascular disease [codes 440–448]). On average across the study and summed across counties, > 333,900 beneficiaries were at risk in our population.

*Data analysis*. We performed time-series analysis to estimate associations between PM_2.5_ sources or constituents and cardiovascular or respiratory hospitalizations by applying a log-linear Poisson regression model:

ln(*E*[*Y_t_^c^*] = ln(*N_t_^c^*) + β*x^c^_t–l_* + α*^c^DOW^t^* + *ns*(*T_t_^c^*,*df_T_*) + *ns*(*D_t_^c^*,*df_D_*) + *ns*(*Ta_t_^c^*,*df_Ta_*) + *ns*(*Da_t_^c^*,*df_Da_*) + *ns*(*t*,*df_t_*) + *I*(*r*), [1]

where

*Y_t_^c^* = hospitalizations in county *c* on day *t*,

*N_t_^c^* = at risk population in county *c* on day *t*,

β *=* coefficient relating pollution to hospitalization rate,

*x^c^_t–l_* = pollution level in county *c* on day *t* at lag of *l* days,

*DOW_t_* = day of week on day *t*,

α*^c^* = coefficient relating day of week to hospitalizations in county *c*,

*ns*(*T_t_^c^*,*df_T_*) = natural cubic spline of temperature in county *c* on day *t* with *df_T_* [degrees of freedom (df) for temperature] = 6,

*ns*(*D_t_^c^*,*df_D_*) = spline of dew point temperature in county *c* day *t* with *df_D_* (df = 3),

*ns*(*Ta_t_^c^*,*df_Ta_*) = spline of average of 3 previous days’ temperature in county *c* day *t* with *df_Ta_* (df = 6),

*ns*(*Da_t_^c^*,*df_Da_*) = spline of average of 3 previous days’ dew point temperature in county *c* day *t* with *df_Da_* (df = 3),

*ns*(*t*,*df_t_*) = spline of time (*t*) with *df_t_* = 8/year (i.e., 8 × 3.5 years = 28), and

*I*(*r*) = indicator of region (coastal for Fairfield or New Haven Counties, inland for Hartford or Hampden Counties).

We considered single-day lags of exposure on the same day as hospitalization (L0), previous day (L1), and 2 days previous (L2). For constituents demonstrating statistically significant associations in single-pollutant models, sensitivity analysis was performed adjusting one at a time for other constituents when the correlation between the second pollutant and the first was < 0.60 in order to avoid collinearity. Results from all analyses represent estimated effects across all four counties. Statistical significance was considered *p*-value < 0.05.

## Results

Table 1 summarizes hospitalizations across all counties (see Supplemental Material, Table S1 for county-level summaries). On average, 73.2 cardiovascular and 26.1 respiratory hospitalizations occurred per day, with the most admissions in New Haven County and the least in Hampden County. The data set contained 95,831 cardiovascular and 34,169 respiratory hospital admissions. Analysis of PM_2.5_ filters for constituents generated 3,273 observation days, whereas the U.S. EPA monitoring network for constituents had 329 observation days for the present study period and time frame. Our data set included constituent data for Hartford County, which had no constituent U.S. EPA monitor during the study period.

**Table 1:** Summary of hospital admissions data.

Admission	Admissions/day			Total admissions across study period
	Mean + SD	Median	IQR	
Cardiovascular	73.2 ± 14.0	73	20	95,831
Respiratory	26.1 ± 9.3	24	10	34,169

Table 2 summarizes estimated PM_2.5_ sources and constituent levels. Daily PM_2.5_ levels averaged 14.0 μg/m^3^ and were highest in New Haven County (average 17.0 μg/m^3^). The regional source, which relates to coal combustion and other factors, on average contributed the largest fraction (40.8%) of PM_2.5_ compared with other sources. Contributions of motor vehicles to PM_2.5_ were similar across counties (26.0–29.7% for any county). Hartford County had a higher percentage of PM_2.5_ from oil combustion (18.2%) than other counties. Correlations between PM_2.5_ sources were low (range, –0.08 to 0.24) (see Supplemental Material, Table S2). Correlations ≥ 0.60 were observed for several pairs of PM_2.5_ constituents, with the highest for Al and Si (0.96).

**Table 2:** Summary of exposure estimates for PM_2.5_ chemical constituents and sources, across all counties.

Constituent/source/temperature	Mean ± SD	Median	IQR	PM_2.5_ total mass (%)
PM_2.5_ (μg/m^3^)	14.0 ± 9.37	11.7	10.7	NA
Al	0.041 ± 0.048	0.0285	0.0353	0.29
BC	1.08 ± 1.000	0.7788	1.32	7.71
Br	0.0018 ± 0.002	0.0014	0.0023	0.01
Ca	0.033 ± 0.027	0.0257	0.0275	0.24
Cl	0.016 ± 0.076	0.0031	0.0079	0.12
Ni	0.0033 ± 0.004	0.0020	0.0033	0.02
K	0.049 ± 0.035	0.0403	0.0333	0.35
S	1.27 ± 1.045	0.9710	0.975	9.07
Si	0.072 ± 0.092	0.0479	0.0625	0.52
Ti	0.0051 ± 0.005	0.0040	0.0043	0.04
V	0.0052 ± 0.008	0.0029	0.0052	0.04
Zn	0.018 ± 0.018	0.0126	0.0150	0.13
Source (μg/m^3^)
Motor vehicle	3.91 ± 4.31	2.53	3.79	28.0
Oil combustion	1.82 ± 2.50	1.07	2.09	13.1
Road dust	1.67 ± 1.93	1.05	1.71	12.0
Regional source	5.69 ± 6.41	3.62	5.34	40.8
Sea salt	0.244 ± 0.92	0.05	0.13	1.75
Temperature
Ambient (^o^C)	49.4 ± 18.2	49.7	30.46	NA
Dew point (^o^C)	40.2 ± 19.1	40.6	30.80	NA
NA, not applicable.				

**Figure 1:**
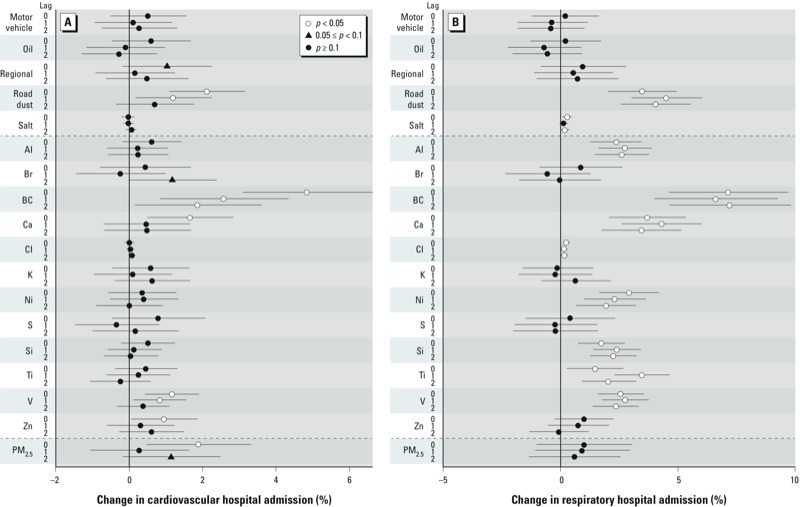
Percent change in risk of cardiovascular (*A*) or respiratory (*B*) hospital admissions per IQR increase in exposure to PM_2.5_ sources, PM_2.5_ total mass, or PM_2.5_ chemical constituents. Data points represent the central estimates, and the horizontal lines represent 95% CIs. IQR values correspond to those in Table 1.

Figure 1 shows effect estimates for PM_2.5_, sources, and constituents for cardiovascular or respiratory hospitalizations according to exposure lag. Central estimates for PM_2.5_ indicate positive associations for both outcomes and all lags, but only the lag 0 association with cardiovascular admissions was statistically significant [1.88%; 95% CI: 0.47, 3.31% for an interquartile range (IQR) increase of 10.7 μg/m^3^]. For PM_2.5_ sources, road dust was significantly associated with respiratory hospitalizations (all lags), with the strongest association estimated for an IQR increase (1.71 μg/m^3^) at lag 1 (4.51% increase; 95% CI: 3.30, 6.01%). Significant associations also were estimated for road dust and cardiovascular admissions (2.11%; 95% CI: 1.09, 3.15% at lag 0) and for sea salt and respiratory admissions (0.27%; 95% CI: 0.08, 0.47% for a 0.13 μg/m^3^ increase at lag 0).

Cardiovascular hospitalizations were significantly associated with BC (all lags), Ca (lag 0, 1.65%; 95% CI: 0.50, 2.82%), V (lags 0 and 1), and Zn (lag 0, 0.95%; 95% CI: 0.05, 1.86%) based on single-pollutant models (Figure 1 and Table 3). For BC and V, associations were strongest for lag 0 (4.83%; 95% CI: 3.08, 6.62% for BC, and 1.16%; 95% CI: 0.43, 1.89% for V).

**Table 3:** Summary of results for co-pollutant adjustment for PM_2.5_ chemical constituents and associations with cardiovascular or respiratory hospital admissions, for constituents with significant associations in single-pollutant models.

Pollutant	Admission	Lag	Single-pollutant effect^*a*^ [% (95% CI)]	Co-pollutant adjustment		
				Robust^*b*^to adjustment by	Not robust^*b*^to adjustment by	Range of central effect estimates (%)
Ca	Cardiovascular	0	1.65 (0.50, 2.82)	Br, Cl, K, Ni, S, Zn	V	1.18–2.01
BC	Cardiovascular	0	4.83 (3.08, 6.62)	Al, Br, Cl, K, Ni, S, Si, Ti, V	NA	4.48–6.00
V	Cardiovascular	0	1.16 (0.43, 1.89)	Al, Br, Ca, Cl, K, S, Si, Ti, Zn	BC	0.39–1.17
Zn	Cardiovascular	0	0.95 (0.05, 1.86)	Cl	Al, Br, Ca, Ni, S, Si, Ti, V	0.44–0.99
Al	Respiratory	1	2.74 (1.62, 3.88)	Br, Cl, BC, Ni, K, S, V, Zn	NA	1.99–4.34
Ca	Respiratory	1	4.31 (2.61, 6.03)	Br, Cl, Ni, K, S, V, Zn	NA	3.13–6.82
Cl	Respiratory	0	0.24 (0.09, 0.39)	Al, Br, Ca, BC, Ni, K, S, Si, Ti, V, Zn	NA	0.19–0.24
BC	Respiratory	2	7.20 (4.64, 9.82)	Al, Br, Cl, Ni, K, S, Si, Ti, V	NA	5.71–9.54
Ni	Respiratory	0	2.92 (1.66, 4.19)	Al, Br, Ca, Cl, K, S, Si, Ti, Zn	BC	1.34–3.21
Si	Respiratory	1	2.41 (1.41, 3.42)	Br, Cl, BC, Ni, K, S, V, Zn	NA	1.70–3.75
Ti	Respiratory	1	3.47 (2.30, 4.65)	Br, Cl, BC, Ni, K, S, V, Zn	NA	2.77–4.19
V	Respiratory	1	2.75 (1.76, 3.75)	Al, Br, Ca, Cl, BC, K, S, Si, Ti, Zn	NA	1.92–2.98
NA, not available. ^***a***^Single-pollutant effect is the increase in risk per IQR increase in pollutant. ^***b***^In this table, associations are considered robust to co-pollutant adjustment if they remained statistically significant; associations are not considered robust to co-pollutant adjustment if they lost statistical significance.						

Respiratory admissions were significantly associated with Al, Ca, Cl, BC, Ni, Si, Ti, and V for all lags (Figure 1 and Table 3). Central effect estimates were highest for lag 1 for most constituents (Al, Ca, Si, Ti, and V), but were largest on the same day (lag 0) for Cl and Ni, and lag 2 had the strongest association for BC.

**Figure 2:**
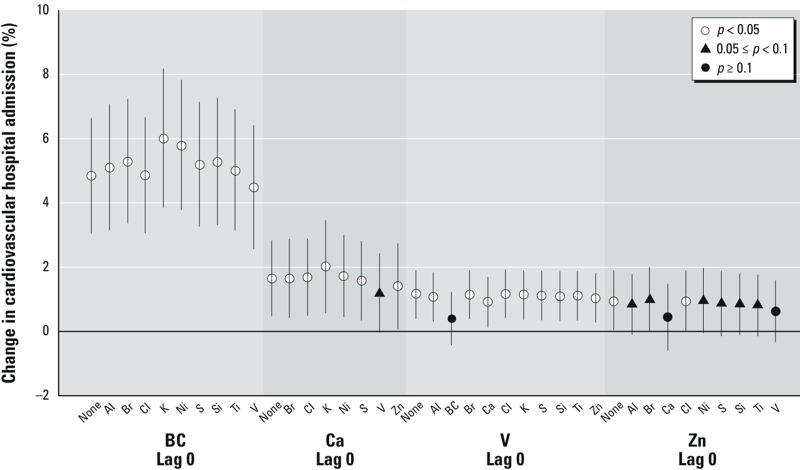
Percent change in risk of cardiovascular hospital admissions per IQR increase in exposure to PM_2.5_ constituent, with adjustment by other PM_2.5_ constituents Ca, BC, V, and Zn. Data points represent the central estimates, and the vertical lines represent 95% CIs. IQR values correspond to those in Table 1.

**Figure 3:**
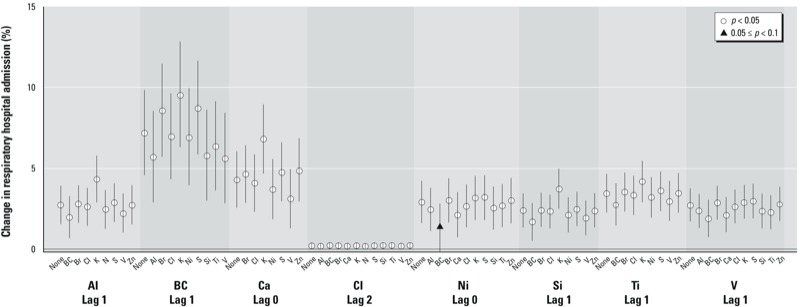
Percent change in risk of respiratory hospital admissions per IQR increase in exposure to selected PM_2.5_ constituents with adjustment by other PM_2.5_ constituents. Data points represent the central estimates, and the vertical lines represent 95% CIs. IQR values correspond to those in Table 1.

We performed sensitivity analyses of co-pollutant adjustment for associations with cardiovascular admissions (Table 3 and Figure 2), and respiratory admissions (Table 3, Figure 3; see also Supplemental Material, Figure S2 where associations between Cl and respiratory hospitalization shown on a narrower *y*-axis scale). In all cases, central effect estimates were in the same direction (i.e., positive associations), and most associations remained statistically significant, with some exceptions (e.g., V adjusted by BC). In particular, the association between same-day Zn and cardiovascular hospitalizations lost statistical significance for most co-pollutant adjustments (central estimate range, 0.44–0.99%).

## Discussion

Same-day PM_2.5_ was significantly associated with cardiovascular, but not respiratory, hospital admissions. Central estimates for PM_2.5_ at lags 1 or 2 for cardiovascular admissions, and all lags for respiratory admissions, were positive but not significant. Previous studies explored PM_2.5_ and hospitalizations for the Medicare population (Bell et al. 2008; Dominici et al. 2006). An earlier study of 202 U.S. counties estimated a 0.86% [95% posterior interval (PI): 0.63, 1.08%] increase in Medicare cardiovascular hospitalizations per IQR increase (for the IQR used in the present study) in same-day PM_2.5_, and a 0.44% (95% PI: 0.09, 0.79%) increase in respiratory hospitalizations for lag 2 PM_2.5_ (Bell et al. 2008). In our four-county study, we estimated a stronger association with cardiovascular admissions (1.88%; 95% CI: 0.47, 3.31% at lag 0) and a higher central estimate for respiratory admissions (0.59%; 95% CI: –1.35, 2.57% at lag 2). However, for counties in the Northeast U.S. region (i.e., the region in which the present study was conducted), the previous study reported associations with PM_2.5_ that were similar to our estimates here (1.16% increase in cardiovascular admissions; 95% PI: 0.85, 1.47% and a nonsignificant 0.30% increase in respiratory admissions; 95% PI: –0.18, 0.78%). Therefore, although the previous study involved a larger study area and somewhat different methods, PM_2.5_ findings were broadly consistent between the two studies.

Recent studies examined PM_2.5_ constituents or sources and hospitalizations or other health outcomes (Bell et al. 2010; Ebisu and Bell 2012; Ostro et al. 2007; Valdés et al. 2012; Zhou et al. 2011). Whereas we estimated positive associations of BC with cardiovascular and respiratory admissions at all lags, in a study of 119 U.S. counties, Peng et al. (2009) reported an association between EC and cardiovascular Medicare admissions only at lag 0, and no association with respiratory admissions. Similarly, we estimated associations between Si and respiratory admissions at lags that were not identified in the national study. In a study of cardiovascular admissions among residents of New York City who were ≥ 40 years of age, Ito et al. (2011) estimated associations with 11 PM constituents, including 6 examined in the present study, and reported statistically significant associations for EC and Zn, but not Ni or Si, consistent with our findings in the present study. However, cardiovascular hospital admissions were significantly associated with Br in the New York City study, in contrast with the present study, and we identified significant associations with V that were not observed in the New York City study. In a previous study of children (≤ 5 or ≤ 19 years of age) in six California counties, Ostro et al. (2009) reported that EC and Si, but not Zn or K, were associated with respiratory hospitalizations. In the present study, we also estimated associations of respiratory admissions with BC and Si, but not Zn or K, in our Medicare population (≥ 65 years of age). Kim et al. (2012) recently reported a significant association between EC and cardiovascular hospitalizations, and a nonsignificant positive association with respiratory hospitalizations, based on constituent data from a single monitoring station in Denver, Colorado.

We estimated significant positive associations between cardiovascular admissions and PM_2.5_ road dust (lag 0 and lag 1) as well as between respiratory admissions and road dust (all lags) and sea salt (lag 0 and lag 2). In contrast, a previous source-apportionment study of PM_2.5_ sources and hospitalizations in New York City (Lall et al. 2011) reported that soil PM_2.5_, which is related to our road dust category, was not associated with respiratory or cardiovascular hospital admissions, except for a significant negative association with cardiovascular admissions at lag 2. In addition, they reported a positive association between traffic PM_2.5_ and cardiovascular admissions, in contrast with null findings for motor vehicle sources and cardiovascular admissions in the present study. However, as in the present study, Lall et al. (2011) did not identify associations between traffic sources PM_2.5_ and respiratory admissions, or associations of residual oil or S with respiratory or cardiovascular admissions.

Zanobetti et al. (2009) examined whether associations between PM_2.5_ mass and hospitalization rates for 26 U.S. communities were modified by the chemical composition of the particles instead of estimating associations between hospitalization and PM_2.5_ constituents or sources directly. The authors reported that higher contributions of Ni and Br strengthened associations between PM_2.5_ mass and cardiovascular hospitalization rates. Our findings were partly consistent, with a significant association between Ni and respiratory admissions but no association of Br with respiratory or cardiovascular admissions. Our results indicated a higher risk of respiratory admissions with higher levels of Ni and no associations for Br.

Our results on chemical constituents add to the body of evidence indicating that some PM_2.5_ constituents and sources are more harmful than others. However, the specific constituents and sources that are associated with adverse health outcomes differ by study. This could relate to differences in populations or study designs, with some studies investigating the health risk of a specific constituent and others investigating how a constituent’s contribution to PM_2.5_ affects PM_2.5_ relative risk estimates or other research questions. The apparent lack of consistency among findings may also relate to heterogeneity of the particle mixture. For example, a given constituent may reflect a different relative contribution of sources in one community than another (e.g., emissions from industry vs. traffic). In addition, the chemical composition of PM_2.5_ from a specific source may differ across cities (e.g., traffic source affected by distribution of vehicle and fuel types and traffic patterns).

Although all of the PM_2.5_ constituents that we studied have multiple sources, several were dominated by specific sources, and were therefore used as source indicators. In the study area, motor vehicles are a main contributor to Zn and BC, road dust to Si and Al, oil combustion to V and Ni, sea salt to Cl, and regional sources to S (Bell et al. 2010). However, in some instances we observed associations with sources but not with their marker constituents. This could relate to uncertainties in source apportionment approaches or measures of constituents, the range of sources for each constituent, and variation in measurement quality. For example, while Al is produced from resuspended soil, other sources of Al include steel processing, cooking, and prescribed burning (Kim et al. 2005; Lee et al. 2011; Ozkaynak et al. 1996; Wang et al. 2005). V is produced from oil combustion but also from the manufacture of electronic products and from coke plant emissions (Wang et al. 2005; Weitkamp et al. 2005). Analysis with PMF may detect associations for sources when marker constituents do not, or vice versa (Ito et al. 2004).

Additional research is needed to further investigate health consequences of PM_2.5_ constituents and sources, including how features of the concentration–response relationship may differ by particle type (e.g., lag structure, seasonal patterns). Other studies have reported seasonal patterns in PM_2.5_ and its associations with hospitalizations (Bell et al. 2008; Ito et al. 2011), but the limited time frame of our data set, and the larger proportion of data collected during the winter than in the summer, prohibited extensive analysis by season. Results may not be generalizable to other locations or time periods. Even in a given location, the chemical composition of PM_2.5_ may change over time due to changes in sources.

Special consideration should be given to exposure methods because spatial heterogeneity differs by constituent or source (Peng and Bell 2010). Use of a smaller spatial unit (e.g., ZIP code) could lessen exposure misclassification. An additional challenge is that key data for particle sources and constituents may be unavailable. For example, our data set did not include organic composition or ammonium sulfate, and the sources identified using our factorization approach might have differed if additional data had been available. Minimum detection limits hindered our ability to estimate exposure for all constituents and to incorporate them in source-apportionment methods. As constituent monitoring networks continue, data will expand with more days of observations being available; however, such data are still substantially less numerous than that for many other pollutants, and not all counties have such monitors.

Particle sources are of key interest to policy makers, but source concentrations cannot be directly measured and must be estimated using methods such as source apportionment, land-use regression, or air quality modeling. Our approach utilized PM_2.5_ filters to provide an expansive data set of constituents for use in source apportionment. This method could be expanded to generate data beyond that of existing monitoring networks, but it requires substantial resources.

Researchers have applied a variety of approaches to estimate how PM_2.5_ constituents or sources affect health outcomes. One of the most commonly applied methods is use of constituent levels (or sources) for exposure, as applied here and elsewhere (e.g., Ebisu and Bell 2012; Gent et al. 2009; Li et al. 2011). Other methods use the constituent’s contribution (e.g., fraction) to PM_2.5_ to estimate associations or as an effect modifier of PM_2.5_ risk estimates (e.g., Franklin et al. 2008), residuals from a model of constituent on PM_2.5_ (e.g., Cavallari et al. 2008), or interaction terms such as between PM_2.5_ and monthly averages of the constituent’s fraction of PM_2.5_ (e.g., Valdés et al. 2012).

Mostofsky et al. (2012) summarized several such modeling approaches, noting that each method has distinct benefits and limitations, and answers different scientific questions. Our approach (constituent levels) has the advantage of results that are readily interpretable, which can aid use of findings in other scientific disciplines and decision making. However, potential limitations include confounding by covarying constituents and PM_2.5_ in situations where PM_2.5_ is associated with the health outcome. Including a variable for PM_2.5_ in the model with the constituent addresses confounding by PM_2.5_ but does not address potential confounding by covarying constituents, and inclusion of such a variable could overadjust if the constituent and PM_2.5_ are correlated (which is likely for constituents representing a large proportion of PM_2.5_ total mass). Methods based on residuals of models of constituents on PM_2.5_ address confounding by PM_2.5_ but produce results that are difficult to interpret and do not estimate relative risk based on the absolute magnitude of a change in constituent level.

The results of various approaches should be interpreted in the context of the scientific question they address and the method’s limitations. Mostofsky et al. (2012) applied six approaches to the analysis of constituents and risk of ischemic stroke onset, and found fairly similar results across methods with the same constituents identified as those with the largest risk estimates. Mostofsky et al. (2012) noted that although effect estimates were not directly comparable across methods, the relative ranking of constituents’ estimates was similar across methods. We applied one of the methods discussed in Mostofsky et al. (2012) to adjust key constituent results by PM_2.5_. Findings were similar to the main results, with identical rankings of central estimates for key results in Table 3 (results not shown).

## Conclusions

Our results contribute to the growing evidence that some particle types are more harmful than others, suggesting that policies aimed at restricting some sources more than others may be more effective for protecting health than is regulating particle mass. As research on air pollution and health moves toward a multipollutant approach (Dominici et al. 2010; Li et al. 2011), policy makers will have better information to develop multipollutant regulations to protect public health. PM_2.5_ levels that meet current regulations may still be harmful if there is no threshold below which PM_2.5_ is not associated with adverse health effects (Anenberg et al. 2010; Brauer et al. 2002) but also if the composition of PM_2.5_ that is below regulatory standards has higher than normal contributions from harmful constituents.

## Supplemental Material

(471 KB) PDF
