# Supplementary material for: Associations of PM2.5 Constituents and Sources with Hospital Admissions: Analysis of Four Counties in Connecticut and Massachusetts (USA) for Persons ≥ 65 Years of Age
Source: Environ Health Perspect. 2013 Nov 8;122(2):138–44. doi: 10.1289/ehp.1306656 (PMC3915260; doi:10.1289/ehp.1306656)

Environmental Health Perspectives doi:10.1289/ehp.1306656

Supplemental Material

Associations of $PM_{2.5}$ Constituents and Sources with Hospital Admissions: Analysis of Four Counties in Connecticut and Massachusetts (USA) for Persons ≥ 65 Years of Age

Michelle L. Bell, Keita Ebisu, Brian P. Leaderer, Janneane F. Gent, Hyung Joo Lee, Petros Koutrakis, Yun Wang, Francesca Dominici, and Roger D. Peng

Table of contents

Supplemental Material, Table S1. Summary statistics of data stratified by county………..…2

Supplemental Material, Table S2. Correlation of $PM_{2.5}$ sources and $PM_{2.5}$ chemical components…………………………………………………………………………........…3

Supplemental Material, Figure S1. Location of monitors and county in Connecticut………...4

Supplemental Material, Figure S2. Percent increase in risk of respiratory hospital admissions per IQR increase in exposure to $PM_{2.5}$ chlorine, with adjustment by other $PM_{2.5}$ components.……..5

Supplemental Material, Table S1. Summary statistics of data stratified by county.

County	Cause of Hospital Admissions	Mean ± SD (admissions /day)	Median (admissions/day)	IQR (admissions /day)	Total admissions across study period	Population at risk/day
Fairfield	CVD	18.8 ± 5.7	18	8	24,672	89,290
	Respiratory	5.6 ± 2.9	5	3	7,324	
Hartford	CVD	21.3±5.9	21	8	27,846	104,558
	Respiratory	8.0 ± 3.7	7	5	10,512	
New Haven	CVD	21.6 ± 6.0	21	9	28,258	93,683
	Respiratory	8.8 ± 4.0	8	5	11,565	
Hampden	CVD	11.5 ± 4.2	11	5	15,055	46,368
	Respiratory	3.6 ± 2.2	3	3	4,768	

Supplemental Material, Table S2. Correlation of $PM_{2.5}$ sources and $PM_{2.5}$ chemical components.

	**$PM_{2.5}$ Sources**					**$PM_{2.5}$ Elements**										
	**Motor vehicles**	**Oil**	**Road dust**	**Sea salt**	**Regional source**	**Al**	**BC**	**Br**	**Ca**	**Cl**	**Ni**	**K**	**S**	**Si**	**Ti**	**V**
**Zn**	0.93	0.40	0.18	0.16	0.02	0.19	0.60	0.49	0.46	0.13	0.54	0.60	0.25	0.20	0.24	0.38
**V**	0.29	0.60	0.28	0.22	0.08	0.25	0.50	0.24	0.39	0.20	0.85	0.28	0.23	0.25	0.21	
**Ti**	0.20	0.04	0.63	0.09	0.19	0.65	0.37	0.16	0.63	0.08	0.22	0.36	0.30	0.65		
**Si**	0.12	0.07	0.89	0.07	0.16	0.96	0.43	0.14	0.81	0.07	0.23	0.44	0.27			
**S**	0.17	0.24	0.16	-0.02	0.95	0.34	0.34	0.38	0.33	-0.02	0.26	0.38				
**K**	0.56	0.39	0.36	0.14	0.20	0.44	0.47	0.52	0.52	0.11	0.39					
**Ni**	0.49	0.67	0.25	0.26	0.06	0.22	0.55	0.37	0.43	0.24						
**Cl**	0.10	0.09	0.03	0.94	-0.07	0.09	0.12	0.14	0.20							
**Ca**	0.42	0.13	0.87	0.20	0.17	0.78	0.66	0.28								
**Br**	0.48	0.38	0.09	0.17	0.25	0.15	0.36									
**BC**	0.61	0.22	0.53	0.12	0.20	0.44										
**Al**	0.11	0.05	0.86	0.08	0.24											
**Regional**	-0.04	0.02	0.06	-0.08												
**Sea salt**	0.13	0.13	0.01													
**Road dust**	0.12	0.00														
**Oil**	0.24															

Supplemental Material, Figure S1. Location of monitors and counties in Connecticut and Massachusetts.

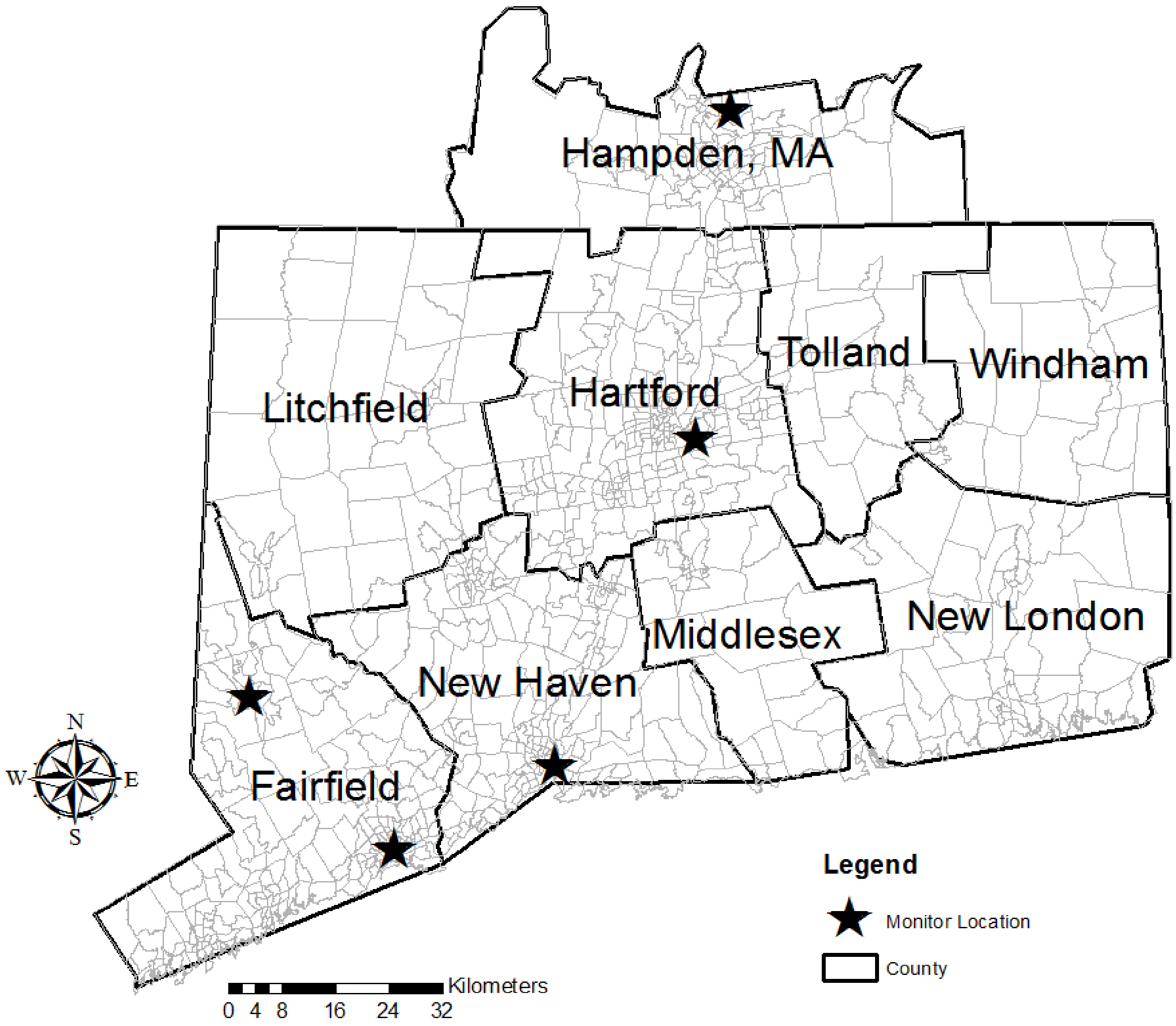

Supplemental Material, Figure S2. Percent change in risk of respiratory hospital admissions per IQR increase in exposure to $PM_{2.5}$ chlorine, with adjustment by other $PM_{2.5}$ components. Note: The point represents the central estimate; the horizontal line represents the 95% confidence interval.

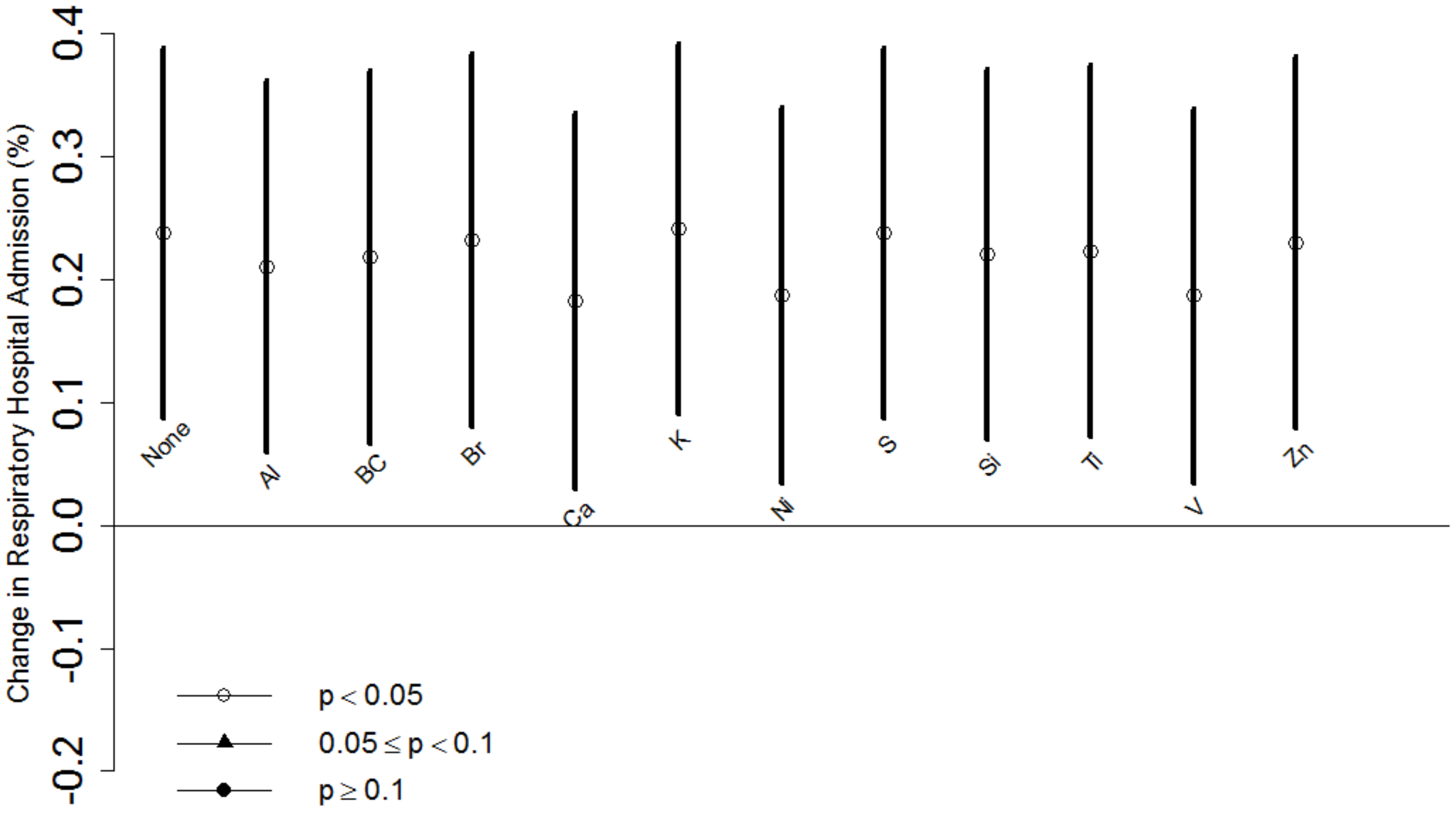

Supplement: (471 KB) PDF [file ehp.1306656.s001.508.pdf]
